# Distinct brain systems are involved in subjective minute estimation with eyes open or closed: EEG source analysis study

**DOI:** 10.3389/fnins.2024.1506987

**Published:** 2024-12-19

**Authors:** Ekaterina Proshina, Dina Mitiureva, Olga Sysoeva

**Affiliations:** ^1^Laboratory of Human Higher Nervous Activity, Institute of Higher Nervous Activity and Neurophysiology of the Russian Academy of Sciences, Moscow, Russia; ^2^Center for Cognitive Sciences, Sirius University of Science and Technology, Sochi, Russia

**Keywords:** time perception, EEG, current source density, sLORETA, duration production task

## Abstract

**Introduction:**

Time perception is a fundamental cognitive function, the brain mechanisms of which are not fully understood. Recent electroencephalography (EEG) studies have shown that neural oscillations in specific frequency bands may play a role in this process. In the current study, we sought to investigate how neurophysiological activity of cortical structures relates to subjective time estimations.

**Methods:**

The study sample included 41 healthy volunteers, who were to produce subjective minutes with eyes closed and open by pressing the response button marking the beginning and end of this time interval. High-density EEG was recorded in parallel and the activity of cortical sources within the theta, alpha, and beta frequency bands was analyzed with standardized low-resolution brain electromagnetic tomography.

**Results:**

The results revealed that activity of several cortical structures within the beta-band correlated with the duration of subjective minutes across participants, which highlights the role of the beta-rhythm in supra-second time perception. The sets of involved structures were different depending on eyes being open or closed, while the produced duration did not differ being around 58 s in both conditions. Individual minute correlated with beta power in the left precuneus, left superior parietal lobule, and right superior frontal gyrus (SFG) during eyes-closed sessions, and with that in the caudal anterior cingulate cortex, cuneus, posterior cingulate cortex, parahippocampal gyrus, and right lingual gyrus during the eyes-open condition. Noteworthy, some structures showed tendencies toward opposite correlations between conditions.

**Discussion:**

Taken together, our findings bridge the gap between functional magnetic resonance imaging and EEG time perception studies and suggest reliance on different aspects of subjective experience when judging about time with eyes open or closed.

## 1 Introduction

Time perception represents a fundamental aspect of human cognition, and it can be defined as the subjective experience or awareness of time passage. It plays a crucial role in our daily lives, affecting a variety of cognitive operations, such as decision-making and motor control. It is suggested that there might be no specific sensory system devoted to time perception but that it is based on integrated sensory information (Wearden, 2016). The study of time perception mechanisms may shed light on the extreme cases of time perception distortions, which are often observed in severe psychopathologies, such as major depression and schizophrenia, and neurodegenerative diseases, such as Parkinson’s disease (Agostino et al., 2003; Yokoe et al., 2009; Thönes and Oberfeld, 2015; Thoenes and Oberfeld, 2017; Buzi et al., 2023; Medvedeva et al., 2023). Advances in neuroimaging methods, such as electroencephalography (EEG) and functional magnetic resonance imaging (fMRI), have enabled exploration of the neural underpinnings of time perception. However, brain mechanisms of time perception remain to be a subject of debate. In the present study, we suggest that time perception relies on a distributed network of structures that are involved depending on the context of time estimation. In other words, we suppose that timing is an emergent property of general neural circuits that also process other types of information, following intrinsic models of time perception mechanisms. Particularly, in line with the Population Clock theory, timing arises from the integration of sensory inputs in a dynamic network, i.e., a population of neurons, whose patterns of activity encode time (Karmarkar and Buonomano, 2007; Buonomano and Laje, 2010; Goel and Buonomano, 2014).

On the level of brain structures, it could be assumed that there are modules or structures more or less specific to time perception with fluctuations of evidence, which depend on the context of a timing task. This view is largely reflected in existing findings, and conceptualizations emerge, such as automatic and cognitively controlled systems, modality-dependent and modality-independent modules. For instance, Macar et al. (2002) reported involvement of attention-related cortical structures, such as the right dorsolateral prefrontal (dlPFC), inferior parietal, and anterior cingulate cortices. Furthermore, they found that the supplementary motor area (SMA) may play a role in processing temporal information, probably through its connections with basal ganglia (Macar et al., 2002). Interestingly, recent stimulation studies have causally confirmed the importance of the right dlPFC for time perception processes (Li et al., 2022; Johari et al., 2023). The automatic system, associated with motor and premotor circuits, and the cognitively controlled system, dependent on the activity of the prefrontal and parietal cortices, were suggested by Lewis and Miall (2003). Resembling results were obtained in a study by Pouthas et al. (2005), where researchers found a number of brain regions involved in time estimation, including the frontal, presupplementary motor area (preSMA) and parietal cortices, as well as the basal ganglia. These regions were predominantly localized in the right hemisphere. Furthermore, the duration of the stimulus was found to be a significant factor, with increased activation observed as the duration increased in a subset of regions, including the preSMA, anterior cingulate cortex, right inferior frontal gyrus, bilateral premotor cortex, and right caudate nucleus (Pouthas et al., 2005). Cumulatively, fMRI studies suggest that brain mechanisms of time perception represent a distributed network of the structures involved with modality-dependent and cross-modal modules (Ivry and Schlerf, 2008; Bueti, 2011; Naghibi et al., 2024).

In the current study, we aimed to investigate brain correlates of time perception using the EEG method. While the majority of studies were conducted with fMRI, EEG also provides valuable information regarding neurophysiological mechanisms of time perception, which is of high significance given that EEG allows us to obtain data directly related to fast neural processes. Furthermore, electrophysiological rhythms, which can be captured via EEG, play a significant role in the functional integration of various brain regions (Buzsáki and Draguhn, 2004), and oscillations of distinct frequencies are associated with specific functional processes (Nunez, 2000; Knyazev, 2007). In EEG studies, time perception has been most commonly investigated using event-related potentials (ERPs) and time-frequency analysis. It has been shown that late positive components time-locked to the ending of an interval encode its subjective and objective duration (Kononowicz and Rijn, 2015; Bueno and Cravo, 2021; Ofir and Landau, 2022; Sysoeva and Vartanov, 2004). As for the EEG rhythms, the importance of prefrontal beta band and parietal alpha/beta frequency range in temporal processing was discovered (Todorovic et al., 2015; Da Silva et al., 2021). Specifically, the alpha-rhythm, which is associated with various cognitive functions including attention and working memory, also plays a role in time perception (Anliker, 1963; Treisman, 1984; Treisman et al., 1994). For example, a variation in parieto-occipital alpha-rhythm power, that are linked to individual difference in time perception, was observed when comparing durations (Rogachev and Sysoeva, 2023). In the right parietal and prefrontal brain regions, alpha and beta band activity was shown to be modulated by the tasks involving temporal expectations and motor timing (Geerligs and Akyürek, 2012; Todorovic et al., 2015; Johari and Behroozmand, 2020). In addition, the beta- and theta-rhythms were found to be relevant for prediction of an interval duration (Kononowicz and Rijn, 2015). In Parkinson’s disease, EEG research has shown that the modulation of beta band neural oscillations during the planning phase of movement is crucial for regulating cortico-striatal neural mechanisms involved in motor timing control (Johari and Behroozmand, 2021). Disruptions to these mechanisms can result in symptoms such as slowed movements. In addition, it was found that in patients with Parkinson’s disease, the overall decrease in beta signal power during timed movement preparation could be explained by changes in dynamics of its desychronization (Heideman et al., 2020). In order to bridge the gap between fMRI and EEG findings, our study was focused on neurophysiological activity of cortical structures in the theta, alpha, and beta frequency bands and how it relates to individual differences in time perception.

On the behavioral level, we employed a duration production task in which participants were to produce 1-min intervals with eyes being either closed or open. The task to produce a 1-min interval is widely used in clinical settings. It was found that the pattern of time estimation distributions showed a noticeable shift according to the level of distress, for example, in cancer patients before the start of chemotherapy (Conev et al., 2019). It was found that depressed individuals tend to overestimate shorter time intervals and underestimate longer ones compared to their non-depressed counterparts (Thönes and Oberfeld, 2015). Considering the connection between depression and serotonin system functioning, it is noteworthy that time perception may be mediated through the serotonergic system (Wittmann et al., 2007; Sysoeva et al., 2010). Therefore, alterations in time perception may not only be evident in individuals with depression but also in healthy subjects who possess genetic predispositions for susceptibility to depression. Consequently, while our study focuses on individuals without health issues, the findings could offer insights into time perception irregularities in individuals with diverse mental health conditions.

We instructed participants to estimate 1-min intervals with their eyes open and closed to investigate the potential influence of functional states associated with external sensory input on time estimation and ongoing brain activity. Assuming that participants maintained consistent attention to the task across both conditions, the primary difference would stem from the availability of external sensory input – specifically visual input, as the environment was silent and devoid of auditory stimuli. This input, being unrelated to the task, could still modulate performance. According to the mechanisms of sensory gating, which regulate the prioritization of sensory inputs (Jones et al., 2000; McCormick et al., 2012), the outcome of duration estimation may vary depending on sensory information used to judge time. For example, with eyes-closed, attention may shift to internal sensory cues, such as interoceptive signals, to estimate the passage of time (Craig, 2009; Wittmann, 2013). Consequently, at the behavioral level, we might also anticipate potential differences in time estimation between the two conditions.

At the neural level, we hypothesize that the activity of distinct brain structures might correlate with task performance, influenced by the availability of visual sensory input. The eyes-closed and eyes-open conditions represent two distinct functional states, characterized by the engagement of different brain networks (Onishi and Hagiwara, 2019; Liu et al., 2020; Weng et al., 2020; Vecchio et al., 2021; Han et al., 2023). These functional states may delineate the set of brain structures comprising the “population clock,” whose dynamic activity underpins time judgment. In other words, depending on the functional state, diverse brain units may contribute to solving the same temporal estimation task. Our primary objective was to analyze the relationship between participants’ subjective time estimates and cortical activity. We further hypothesize that specific brain regions may influence the tendency to overestimate or underestimate the 1-min interval, corresponding to a deceleration or acceleration of subjective time, respectively.

Overall, this study sought to investigate the relationship between neurophysiological activity in cortical structures and time perception, specifically through connection to between-individual variations of 1-min interval production. While prior fMRI and brain stimulation studies suggest that this process predominantly involves right prefrontal and parietal regions (Wiener et al., 2010; Cona et al., 2021; Li et al., 2022; Johari et al., 2023; Naghibi et al., 2024), we adopted an exploratory approach and used standardized low-resolution brain electromagnetic tomography (sLORETA) to analyze current source density (CSD) in the theta, alpha, and beta bands. This decision reflects the novelty of using EEG to assess cortical sources during subjective minute production under eyes-open and eyes-closed conditions. By presenting all statistically significant effects, we aim to provide a foundation for future research to refine hypotheses and build upon our findings. We hypothesize that the neural mechanisms underlying time perception might differ between the eyes-open and eyes-closed conditions, with certain brain regions associated with either overestimation or underestimation of time.

## 2 Materials and methods

### 2.1 Participants

The study sample consisted of 41 individuals (34 females, mean age 26.7, SD = 7.18 and 7 males, mean age 26.7, SD = 5.71). The age range of the whole sample was from 18 to 42 years. According to the power analysis conducted using the G*power software (Faul et al., 2007), the sample size exceeds the minimally required sample size (*N* = 29) to detect a medium effect in correlational analysis (with *R* of 0.5, a two-tailed test, alpha error probability of 0.05 and 80% power).

Participants in the study were screened for any mental, neurological, or serious somatic diseases, recent head injuries, or taking psychotropic drugs. Additionally, they were asked to refrain from smoking or drinking coffee for a few hours prior to the study. All applicable subject protection guidelines and regulations were followed in conducting the research in accordance with the Declaration of Helsinki. All participants signed written informed consent. The study protocol was approved by the Ethical Commission of the Institute of Higher Nervous Activity and Neurophysiology of the Russian Academy of Sciences (Ethics protocol: No. 2, 30 April 2021).

### 2.2 Time perception measurements

**FIGURE 1:**
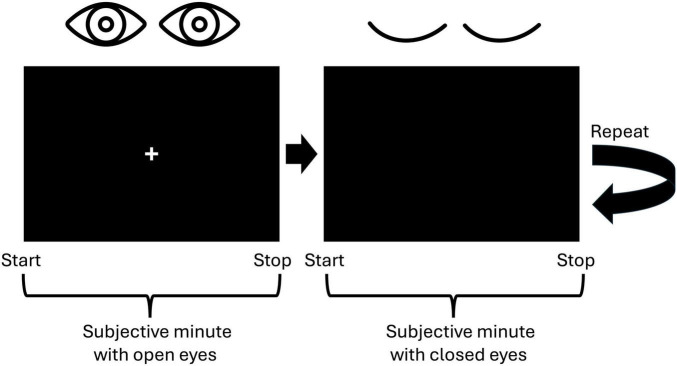
Visualization of the experimental paradigm. Participants were to indicate the start and the stop of a subjective minute by pressing the spacebar. First, they were to produce a 1-min interval with open eyes and then with closed eyes. This procedure was repeated one more time resulting in two trials with open eyes and two trials with closed eyes.

We employed the Subjective Minute Test, a variation of the duration production task designed to assess time perception by requiring participants to estimate an interval of 1 min. This test was programmed using Presentation software (Neurobehavioral Systems, Inc., Berkeley, CA, USA, www.neurobs.com), and was conducted alongside EEG recordings. Participants were instructed to produce a minute-long interval four times, alternating between open and closed eyes. Specifically, they completed two trials with their eyes open and two with their eyes closed. To initiate the task, participants pressed the spacebar, signaling the start of the minute, followed by the appearance of a cross on the screen. Then they pressed the spacebar a second time when they believed a minute had elapsed (Figure 1). Participants were explicitly instructed to refrain from using any external aids, such as tapping or counting, relying solely on their internal sense of time.

When selecting the number of trials, we adhered to the fact that a duration starting from 20 s is appropriate for analyzing resting-state activity (Möcks and Gasser, 1984). The alternation between open and closed eyes aimed to mitigate drowsiness during the closed-eye intervals (Maltez et al., 2004). This approach yielded EEG recordings consisting of two trials under the open-eye condition and two under the closed-eye condition, presented in an alternating sequence. The length of the EEG segment for each condition varied according to the individual participant’s responses.

### 2.3 EEG recording and preprocessing

During the experiment, 64-channel EEG was acquired. The electrodes were positioned according to the 10-10 international system. This number is generally sufficient to source reconstruction as can be referred from previous studies (Michel et al., 2004). BrainProduct amplifier with a 0.1–100-Hz analog bandpass filter was used for signal amplification. The sampling rate was 500 Hz. Electrode impedances were kept at or below 15 kΩ. Due to the active electrode technology, the recording system we use can record high quality EEG data with higher impedances (up to 25–50 kΩ). The AFz electrode was used as a ground and the Cz as a reference. A finite impulse response (FIR) filter was used with a frequency range of 1–30 Hz. Bad channels were manually selected and interpolated. The data were recomputed to the average reference. Artifact correction was performed using the independent component analysis (ICA) method (FastICA algorithm, 30 components). Artifacts associated with eye blinks, and movements were removed. The selection of components adhered to the recommendations provided by the ALICE toolbox (Soghoyan et al., 2021). EEG data were preprocessed and analyzed using MNE-python (Gramfort, 2013).

### 2.4 EEG analyses

The EEG segments were cropped according to the shortest subjective minute production in the sample (∼30 s). Preprocessed EEG segments corresponding to open and closed-eye conditions (60 s each) were divided into 2.5-s epochs with a 50% overlap. Epochs containing artifacts were automatically discarded if their peak-to-peak amplitude surpassed a threshold of 180 μV. The mean number and the standard deviation of epochs were 45.2 ± 1.6 per condition per participant.

We investigated cortical sources activity within the following frequency ranges: theta (4–7 Hz), alpha (8–12 Hz), and beta (13–30 Hz). The current study focuses on identifying the locations and characteristics of brain activity through measurements obtained from electrodes positioned on the scalp. This undertaking is linked to the EEG “inverse problem,” which emerges from the fact that multiple source configurations can produce identical EEG signals, complicating the accurate identification of source locations without additional information or assumptions regarding the sources. As a result, various algorithms have introduced different assumptions and constraints to tackle this issue. Among these approaches, sLORETA (Pascual-Marqui, 2002) is particularly notable as a robust method. It is a non-parametric technique that estimates the distribution of electrical activity in the brain while implementing constraints that enhance spatial resolution and increase stability against noise. sLORETA has demonstrated efficacy in numerous cognitive studies (e.g., Dyson et al., 2010; Ocklenburg et al., 2012; Nguyen et al., 2014). Furthermore, sLORETA is recognized for having the minimal localization error for test sources (Pascual-Marqui, 2002).

A forward solution model was calculated based on the 10-10 montage and averaged brain MRI data (MNE “fsaverage” dataset). The boundary element model (BEM) (see, e.g., Mosher et al., 1999 was constructed using the linear collocation method with the isolated skull approach (Hamalainen and Sarvas, 1989). The default MNE electrical conductivities were applied (0.3 S/m for the brain and the scalp, and 0.006 S/m for the skull) (Gramfort et al., 2014). The source space was computed with an “oct6” resolution, which leads to 4,098 vertices per hemisphere with an average distance of ∼5 mm between them. The resulting CSD distribution was separated into time series for 68 cortical structures (regions of interest, ROI) according to Destrieux Atlas (Freesurfer “aparc” parcellation). The CSD estimations were averaged over epochs, as the temporal dynamics of the signal were not of interest. As a result, mean sLORETA CSD value was obtained for each of 68 ROI.

### 2.5 Statistical analysis

Spearman’s test was employed to calculate the correlation between the subjective minute duration and the CSD during the task in both close and open eye states (*p* < 0.05). In addition, the accuracy of subjective minute production was assessed as a modulus of the difference between a subjective estimation and the objective minute. The Benjamini–Hochberg correction was used to control the multiple comparisons problem (Benjamini and Hochberg, 1995). This method is designed to control the false discovery rate (FDR), which is the expected proportion of false discoveries among the rejected null hypotheses. All calculations were performed by means of the free R software packages.

## 3 Results

Descriptive statistics for subjective minute duration showed that the mean duration was similar for both conditions (eyes-open: 58.62 s, eyes-closed: 57.73 s), with slightly higher standard deviation in the eyes-closed condition (Table 1). The Wilcoxon test did not reveal any statistically significant differences between conditions (*p* = 0.5).

**TABLE 1:** Descriptive statistics of samples’ subjective minute duration.

	Mean	SD	Min	Max
Opened eyes	58.62	17.92	28.73	97.04
Closed eyes	57.73	20.51	26.71	104.21

**FIGURE 2:**
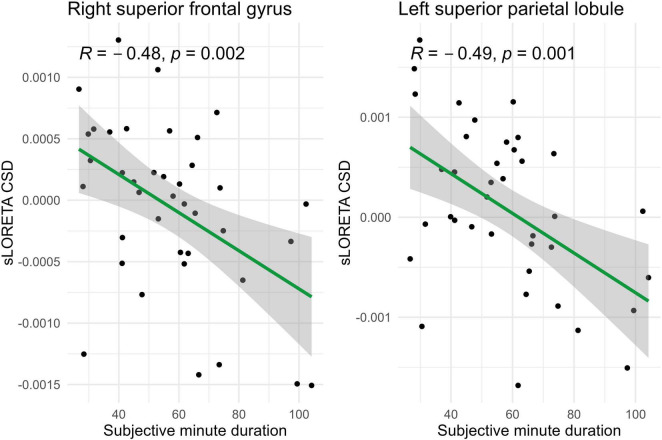
Significant Spearman correlations (*p* < 0.05) between right superior frontal gyrus/left superior parietal lobule CSD and subjective minute duration.

**FIGURE 3:**
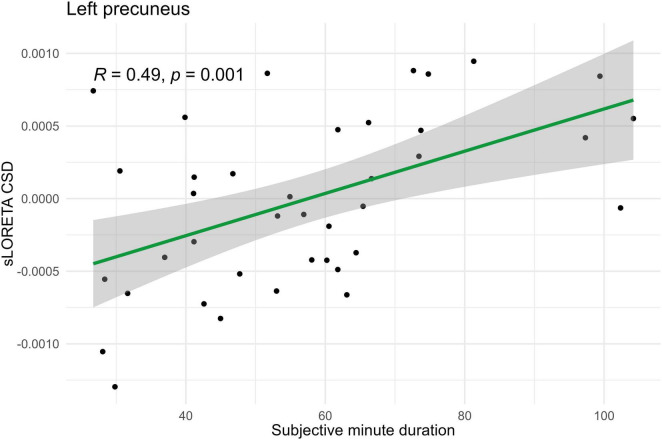
Significant Spearman correlation (*p* < 0.05) between left precuneus CSD and subjective minute duration.

**FIGURE 4:**
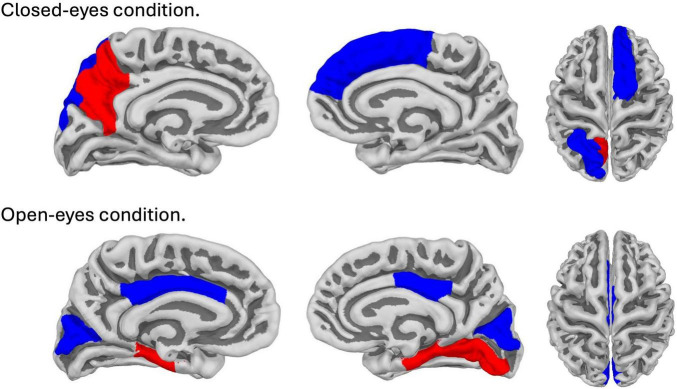
Brain map with areas showing significant correlations (*p* (corr.) < 0.05) with subjective minute duration. The top line corresponds to the closed-eyes condition; the bottom line corresponds to the open-eyes condition. Red color indicates areas showing positive correlation, blue color indicates areas showing negative correlation.

Spearman correlations were calculated between subjective minute duration and sLORETA CSD for different brain regions and frequency bands. All of the effects identified were associated with the beta band. In the eyes-closed condition, significant positive correlations were found between beta band activity in the left precuneus and subjective minute duration. Significant negative correlations were observed between beta band activity in the right SFG and left superior parietal lobule with subjective minute duration (Table 2 and Figures 2–4).

**TABLE 2:** Significant Spearman correlations between subjective minute duration and sLORETA CSD by ROI (eyes-closed), beta frequency range.

ROI	*R*s	*p*-Value
Precuneus lh	0.494	0.036
Superior frontal rh	−0.482	0.036
Superior parietal lh	−0.487	0.036

The Benjamini–Hochberg method controls the FDR. Correlations are significant at the 0.05 level (two-tailed). lh, left hemisphere; rh, right hemisphere.

**FIGURE 5:**
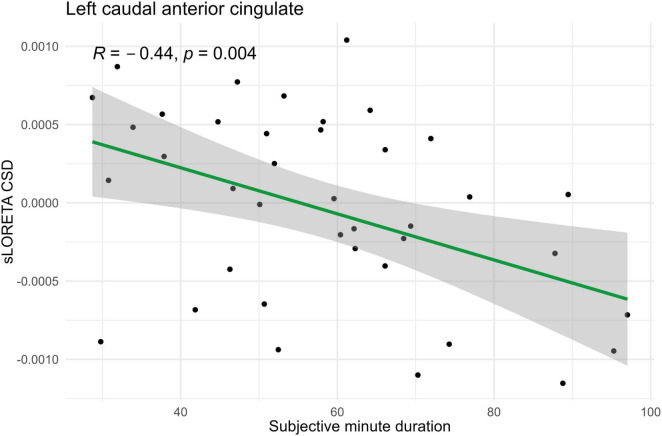
Significant Spearman correlation (*p* < 0.05) between left caudal anterior cingulate CSD and subjective minute duration.

**FIGURE 6:**
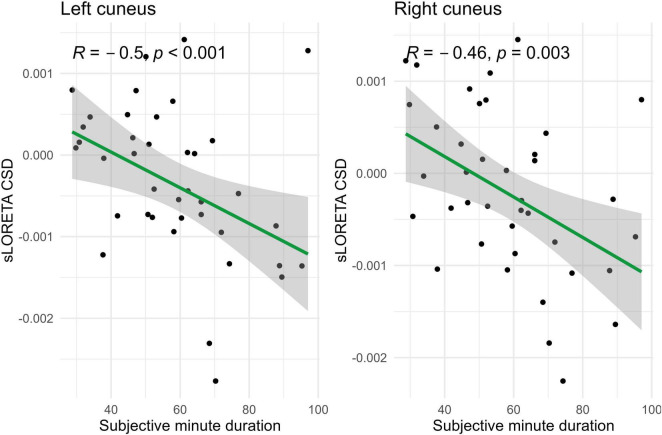
Significant Spearman correlations (*p* < 0.05) between left and right cuneus CSD and subjective minute duration.

**FIGURE 7:**
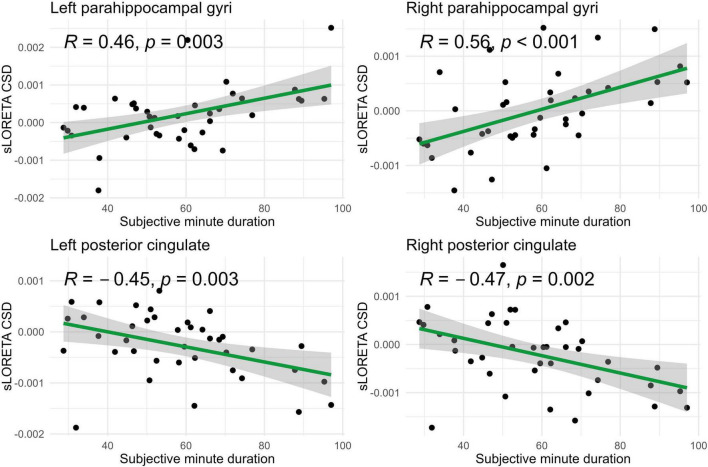
Significant Spearman correlations (*p* < 0.05) between right/left posterior cingulate and parahippocampal gyri CSD and subjective minute duration.

In the eyes-open condition, a greater number of brain regions exhibited a significant correlation with the subjective minute duration. Negative correlations were found between beta band activity in the left caudal anterior cingulate, left and right cuneus, left posterior cingulate, and right posterior cingulate. Positive correlations were observed between beta band activity in the right lingual gyrus and left and right parahippocampal gyri with subjective minute duration (Table 3 and Figures 4–7).

**TABLE 3:** Significant Spearman correlations between subjective minute duration and sLORETA CSD by ROI (eyes-open), beta frequency range.

ROI	*R*s	*p*-Value
Caudal anterior cingulate lh	−0.442	0.036
Cuneus lh	−0.502	0.032
Cuneus rh	−0.461	0.032
Lingual rh	0.476	0.032
Parahippocampal lh	0.458	0.032
Parahippocampal rh	0.557	0.012
Posterior cingulate lh	−0.453	0.032
Posterior cingulate rh	−0.467	0.032

The Benjamini–Hochberg method controls the FDR. Correlations are significant at the 0.05 level (two-tailed). lh, left hemisphere; rh, right hemisphere.

In examining the correlation between similar regions within states, it was revealed that the effect was different in eyes-opened and eyes-closed conditions. The correlation coefficient (*R*s) was opposite in the same brain areas (Table 4). In addition, the observed correlations were found only in relation to duration of a produced minute, but not with the accuracy of this production (taken as a modulus of the error, Supplementary Table 1).

**TABLE 4:** Correlations between subjective minute duration and sLORETA CSD in the beta frequency range.

	Eyes-open			Eyes-closed		
**ROI**	***R*s**	***p*-Value uncorrected**	***p*-Value BH-corrected**	***R*s**	***p*-Value uncorrected**	***p*-Value BH-corrected**
Caudal anterior cingulate lh	−0.442	0.004	0.036*	0.191	0.238	0.558
Cuneus lh	−0.502	<0.001	0.032*	0.300	0.059	0.252
Cuneus rh	−0.461	0.003	0.032*	0.300	0.059	0.252
Lingual rh	0.476	0.002	0.032*	−0.267	0.096	0.336
Parahippocampal lh	0.458	0.003	0.032*	−0.145	0.37	0.674
Parahippocampal rh	0.557	<0.001	0.012*	−0.077	0.636	0.84
Posterior cingulate lh	−0.453	0.003	0.032*	0.269	0.093	0.336
Posterior cingulate rh	−0.467	0.002	0.032*	0.310	0.051	0.249
Precuneus lh	−0.337	−0.033	0.15	0.494	0.001	0.036*
Superior frontal rh	0.415	0.008	0.058	−0.482	0.002	0.036*
Superior parietal lh	0.255	0.109	0.246	−0.487	0.001	0.036*

rh, right hemisphere; lh, left hemisphere; BH, Benjamini–Hochberg correction.

*p-value BH-corrected < 0.05.

## 4 Discussion

The aim of this work was to investigate associations between subjective time judgments, i.e., production of the subjective minute with eyes closed or open, and neurophysiological activity of cortical brain structures in the theta, alpha, and beta frequency bands. Notably, all of the associations were identified within the beta frequency range, with different brain regions showing either negative or positive correlations, also depending on the eyes being open or closed.

While no behavioral differences were observed in subjective minute production between the eyes-open and eyes-closed conditions, distinct cortical structures were implicated under each condition. Interestingly, some brain regions exhibited statistical tendencies toward opposite effects between the two conditions, suggesting functional state-dependent neural dynamics. Therefore, this implies that time perception, as an emergent property of distributed neural networks, can rely on different functional states of the brain to produce similar behavioral outcomes. Such functional flexibility of the brain mechanisms involved supports the intrinsic models of time perception, particularly theories suggesting that timing relies on general-purpose neural circuits rather than a dedicated, centralized timing mechanism (Karmarkar and Buonomano, 2007; Ivry and Schlerf, 2008; Buonomano and Laje, 2010).

Additionally, these observations suggest that diverse aspects of subjective experience could be recruited to judge about the time under these conditions. Importantly, the observed correlations were found in relation to duration of a produced minute, but not with the accuracy of this production (taken as a modulus of the error). Given this result, we will discuss the findings in terms of subjective time acceleration or deceleration, rather than the accuracy of a learned interval production.

It is important to acknowledge that our study did not reveal any significant findings regarding alpha and theta frequency bands. Given the range of existing data on their contribution to temporal processing, our results are in line with a number of previous studies suggesting that their contribution is thought to be complex and multifaceted (Van Viegen et al., 2017). For example, the effects found in previous studies for alpha frequency band involved comparing auditory or visual stimulus from sub-second ranges to 6-s ranges (Van Viegen et al., 2017; Rogachev and Sysoeva, 2023), but our study involved a prolonged focus on the time course, which was furthermore modulated by eye-open and eye-closed states in which neural networks are externally and internally oriented.

### 4.1 The role of beta oscillations in time perception

The involvement of numerous brain regions and differences between eye-open and eye-closed conditions may indicate that beta activity facilitates the integration of sensory information, attention, and memory, thereby shaping our subjective experience of time. The obtained results are of interest in light of extensive recent research on the role of beta oscillations in time perception. Initially, alpha oscillations were identified as a potential contributor to time perception (Anliker, 1963; Treisman, 1984; Treisman et al., 1990). Subsequently, the role of other brain rhythms in this process was explored, yet the findings were not always consistent as it soon became apparent that the experimental design shapes the outcomes of such studies. This issue is thoroughly explained by Wiener and Kanai (2016). They examined the role of different frequency bands and regions in time perception and highlighted that temporal processing in the brain is an adaptable mechanism, which induces dynamic interactions among brain structures and networks (Wiener et al., 2011; Wiener and Kanai, 2016). Following this, Wiener et al. (2018) determined that the beta-rhythm power might reflect a shift in the value of the memory standard over time. Using transcranial alternating current stimulation (tACS), they observed that beta-rhythm stimulation of the fronto-central cortex leads to perception of visual stimuli as lasting longer. As a result, the researchers inferred the critical role of beta oscillations in encoding and retaining memories of temporal intervals (Wiener et al., 2018).

In studying the function of beta oscillations, the problem of distinguishing between motor and perceptual aspects of timing arises. Time perception tasks often imply conducting a movement to indicate a task-relevant response. Frank (2011) emphasizes the difficulty of separating temporal and motor components given the interaction of cognitive and motor circuits in the corticostriatal loop. Tasks that necessitated motor system involvement were labeled as “motor” timing, whereas those that did not were referred to as “perceptual” timing. The use of motor timing paradigms in non-human primates has revealed that putamen-originating beta power is larger for longer durations (Bartolo et al., 2014; Bartolo and Merchant, 2015). However, Kononowicz and Rijn (2015) hypothesized that if beta power dynamics were solely linked to motor sequence generation, fluctuations in beta power would not correlate with behavior on a time production task. At the same time, if beta power were linked to the internal sense of time, it should correlate with the length of the produced interval. The authors conclude that the initial motor inhibition at the onset of an interval affects the duration of produced intervals. This suggests that beta oscillations play a role in interval timing beyond just motor preparation (Kononowicz and Rijn, 2015). The problem of the influence of motor response and the impact of stimulus predictability on the outcomes is also discussed in detail in Wiener et al. (2018).

Thus, there is more and more evidence in favor of the existence of a division into cognitively controlled timing and motor timing, which are probably characterized by the involvement of different brain regions. In our study, we used the cognitively controlled timing paradigm, which presupposes the use of supra-second intervals (the Subjective Minute Test), which assumes a production of an interval of about 60 s. The data analysis was carried out by cutting the EEG into epochs, which then were averaged; therefore, we can claim a general absence of a motor component in our results. Nevertheless, the only frequency range in which we observed correlations with the duration of the subjective minute—within our analysis of the alpha, beta and theta bands—was the beta range. A MEG study conducted by Kulashekhar et al. (2016) employing a non-motor paradigm, revealed that comparing durations led to notably increased beta power amplitudes in the parietal-frontal network, which includes the SMA, while encoding stimulus durations. Moreover, elevated beta amplitudes were linked to both correct and incorrect selections, highlighting their substantial behavioral importance (Kulashekhar et al., 2016). These findings, when considered alongside our own, indicate that the beta range is likely to be associated not only with motor aspects but also with the cognitive components of time perception.

### 4.2 Brain areas associated with subjective minute production

During eyes-closed sessions, beta activity of the left precuneus correlated positively with subjective minute duration, meaning higher beta power was linked to longer subjective minutes and deceleration (dilation) of subjective time. Beta activity in the right SFG and left superior parietal lobule showed negative correlations, indicating higher beta power association with shorter subjective minutes and acceleration (compression) of subjective time. The right SFG exhibited a tendency for relation of another direction with subjective minutes during the open-eye condition.

The SFG includes subregions which are often reported to be engaged in temporal processing, such as the SMA and dorsolateral part of the prefrontal cortex (PFC). It is well-known that the SMA plays a crucial role in temporal processing (Casini and Vidal, 2011; Schwartze et al., 2012; Naghibi et al., 2024). According to a recent meta-analysis of fMRI studies, the SMA is one of the structures which is consistently involved in time perception across diverse experimental paradigms and presentation modalities (Naghibi et al., 2024). The SMA’s role in time perception aligns with embodied cognition theories, which propose that bodily states generated through action can serve as anchors for cognitive processing. This suggests that motor activity, including that in the SMA, may represent an active form of perception, structuring the perception of time through sensorimotor integration and top-down predictions (Fernandes and Garcia-Marques, 2019). While the SMA may represent motor derivatives in time perception, not specifically sensitive to the time scale, the PFC is involved in cognitively controlled timing mechanisms for longer durations, which are tightly linked with working memory and attention (Coull et al., 2011). It has been demonstrated that the disruption of the right PFC activity with transcranial magnetic stimulation (TMS) leads to impairment of supra-second timing (Jones et al., 2004; Koch et al., 2007), and there is causal evidence, obtained with transcranial direct current stimulation, of the involvement of the right dlPFC in time perception (Li et al., 2022; Johari et al., 2023).

The joint activity of the right SFG and parietal regions (superior parietal left – lateral part and precuneus as a medial part) represents “cognitively controlled” timing system, which is systematically observed in neuroimaging time perception studies (Lewis and Miall, 2003; Üstün et al., 2017; Naghibi et al., 2024). In addition, parietal regions are implicated in multisensory integration for body positioning and movement in space. There is evidence that within these areas the convergence between the space and time representations occurs, and the underlying mechanism could be cross-modal magnitude encoding (Walsh, 2003; Buzsáki and Llinás, 2017). Another point is that the sensory integration occurring in these regions is key to represent the bodily self. With intracranial stimulation, it was shown that disruption of anterior precuneus leads to loss of the bodily physical self accompanied with feeling of falling, dropping, and dizziness, and the superior parietal lobule showed functional connections with this “hot zone” (Lyu et al., 2023).

Together the left SPG, right SFG, and left precuneus comprised the core “closed-eyes” time estimation network although the left precuneus and right SFG had a tendency for an effect of different direction during the eyes-open condition. Their coherent involvement specifically during the eyes-closed condition could be interpreted as an inward appeal to internal sources of information to judge about time passage. In line with the known functions of these structures, these internal sources of information could be related to spatial-time representations of a bodily self according to an embodied framework (Naghibi et al., 2024).

During eyes-open sessions, beta activity exhibited negative correlations with subjective minute duration in the left caudal ACC, bilateral cuneus, and bilateral PCC. This suggests that higher beta power in these regions was related with shorter subjective minutes and acceleration (compression) of subjective time. Positive correlations between the beta activity and subjective minute duration were found in the right lingual gyrus and bilateral parahippocampal gyrus. Therefore, higher beta-rhythm power in these areas was associated with longer subjective minutes and deceleration (dilation) of subjective time. In addition, the PCC and cuneus exhibited tendencies toward inverse relations with subjective minutes in the eyes-closed condition.

In contrast to the eyes-closed condition, the set of structures which showed relationships with a subjective minute during the eyes-open condition might indicate attentional focus to external sources of information to judge about time passage. For instance, a combination of the caudal ACC, bilateral cuneus, and right lingual gyrus involvement could point to activation of the mechanisms of visually guided attention (Menon, 2015). The bilateral cuneus and lingual gyrus are components of a visual network, thereby indicating activity of modality-dependent temporal mechanisms (Bueti, 2011). The caudal ACC is relatively rarely reported to be engaged in time perception processes but its role in supra-second timing is highlighted in several fMRI studies, which is usually interpreted as attentional control or decision-making derivative (Pouthas et al., 2005; Üstün et al., 2017).

The PCC and parahippocampal gyrus are the nodes of the default mode network (DMN) (Zhao et al., 2022). Being one of the most metabolically active cortical regions both at rest and during cognitive tasks, the PCC possesses various functions including pain processing, episodic memory retrieval, and conscious awareness (Raichle et al., 2001; Leech and Sharp, 2014). It was found that in the context of time perception, the PCC stands for the aspects related to working memory (Üstün et al., 2017). The parahippocampal gyrus is also involved in working memory processes. However, while the PCC is responsible for retrieval, the parahippocampal gyrus may underlie the integrative and maintenance functions (Luck et al., 2010; Ward et al., 2014). Taken together, the activity of these structures points to involvement of working memory to encode, maintain, and retrieve perceptual moments, which constitute subjective time.

### 4.3 Limitations

Among the limitations of the study, it is important to mention that EEG possesses a relatively low spatial resolution; thus, we analyzed the activity of cortical sources only. However, the subcortical structures, such as the basal ganglia, also play a crucial role in time perception. Another limitation of this study is its correlational design, which prevents establishing causal links between neural activity and time perception. Future research using causal methods, such as brain stimulation, is needed to confirm the functional roles of the identified brain regions. In addition, we could not completely rule out the influence of the motor component in our study, although we believe that it was largely eliminated by the peculiarities of the paradigm, which involved pressing only once at the beginning and end of the subjective minute, and by the division of the data into epochs with subsequent averaging.

## 5 Conclusion

The present study revealed correlation between neurophysiological activity of cortical sources and time perception. Specifically, the beta-rhythm power of several cortical structures during production of a subjective minute was associated with its duration. These included the right SFG, left superior parietal lobule, left precuneus, left caudal ACC, bilateral cuneus, bilateral PCC, right lingual gyrus and bilateral parahippocampal gyrus. Our results correspond with those obtained with fMRI in terms of localization, bridging the gap between the fMRI and EEG findings, and also highlight the role of beta-rhythm in supra-second time perception. The set of the recruited structures also depended on eyes being closed or open during task completion. This observation suggests involvement of diverse brain networks and, probably, reliance on distinct aspects of subjective experience while judging time under these conditions.

## Data Availability

The data related to the project is accessible through the OSF link, https://osf.io/myp4t/?view_only=63ff3cc6857541a7a18d9f7b caf69796.
