# Supplementary material for: Distinct brain systems are involved in subjective minute estimation with eyes open or closed: EEG source analysis study
Source: Front Neurosci. 2024 Dec 19;18:1506987. doi: 10.3389/fnins.2024.1506987 (PMC11693652; doi:10.3389/fnins.2024.1506987)
Supplement: Supplementary file 1 [file Table_1.docx]

Supplementary Material

Table A1. Results of correlation test with the accuracy of time interval estimation by brain area, BH corrected

|  | eyes-open | eyes-closed |
| --- | --- | --- |
| ROI | p-value | |
| caudal anterior cingulate.lh | 0.987 | 0.78 |
| cuneus.lh | 0.987 | 0.87 |
| cuneus.rh | 0.987 | 0.93 |
| lingual.rh | 0.987 | 0.93 |
| parahippocampal.lh | 0.987 | 0.93 |
| parahippocampal.rh | 0.987 | 0.951 |
| posterior cingulate.lh | 0.987 | 0.93 |
| posterior cingulate.rh | 0.987 | 0.93 |
| precuneus.lh | 0.987 | 0.9 |
| superior frontal.rh | 0.987 | 0.972 |
| superior parietal.lh | 0.987 | 0.87 |
